# Food-Related Health Emergency-Disaster Risk Reduction in Rural Ethnic Minority Communities: A Pilot Study of Knowledge, Awareness and Practice of Food Labelling and Salt-intake Reduction in a Kunge Community in China

**DOI:** 10.3390/ijerph16091478

**Published:** 2019-04-26

**Authors:** Emily Ying Yang Chan, Holly Ching Yu Lam, Eugene Siu Kai Lo, Sophine Nok Sze Tsang, Tony Ka Chun Yung, Carol Ka Po Wong

**Affiliations:** 1 Collaborating Centre for Oxford University and CUHK for Disaster and Medical Humanitarian Response (CCOUC), JC (Jockey Club) School of Public Health and Primary Care, Faculty of Medicine, Chinese University of Hong Kong, Hong Kong, China; hollylam@cuhk.edu.hk (H.C.Y.L.); euglsk@cuhk.edu.hk (E.S.K.L.); sophinetsang@cuhk.edu.hk (S.N.S.T.); yungtony@cuhk.edu.hk (T.K.C.Y.); wongcarol@cuhk.edu.hk (C.K.P.W.); 2 Nuffield Department of Medicine, University of Oxford, Oxford OX3 7BN, UK; 3 François-Xavier Bagnoud Center for Health & Human Rights, Harvard University, Boston, MA 02138, USA

**Keywords:** food-related health, risk reduction, rural, ethnic minority, food label, salt, food safety, education intervention, Health-EDRM

## Abstract

Food safety and unhealthy dietary pattern are important global health problems. Understanding food-related health needs and providing corresponding support are important to health risk reduction. A needs assessment, education intervention for food labelling, and another intervention for salt-intake reduction were conducted in a rural Kunge community in Yunnan, China in 2014, 2015 and 2016, respectively. Not checking the expiry date of packaged food (37.1%) and a high salt diet (53.9%) were the most common problems in the community. Both topics were selected for education intervention. Pre- and post-intervention questionnaires were used to evaluate the effectiveness. Education interventions were found effective in improving food-health-related knowledge, changing attitudes toward behaviors such as willingness to read food labels before buying and consuming packaged food. However, no significant improvements were found for the attitudes toward not consuming expired food, controlling salt-intake, and decreasing the consumption of cured food. Health education was shown to be effective in promoting food-health-related knowledge but was limited in changing relevant behaviors in a rural ethnic minority community.

## 1. Introduction

Globally, food safety and unhealthy dietary have always been major health concerns for societies. According to World Health Organization (WHO) [1], food safety is the “assurance that food will not cause harm to the consumer when it is prepared and/or eaten according to its intended use”. It has been a persistent problem in China and has caused different foodborne illnesses and food poisoning incidents over-time [2]. In addition, inappropriate dietary behavior is also prevalent in China, leading to significant rates of non-communicable diseases. For example, high salt consumption is associated with hypertension, cardiovascular disease and strokes [3]. This study focuses on two food-related health topics: food labelling and salt consumption in rural China population with education intervention.

Food safety issues are often multifaced. For example, suboptimal food safety system (for example regulation violations during transportation or storage) could lead to large scale food-borne disease outbreaks in a community. On the individual level, insufficient food safety knowledge could lead to incorrect food-process or consumption and to food-borne illness eventually [4,5]. Relevant food-related knowledge, awareness, and practices vary from place to place and were shown to be different between urban and rural [6]. A study from Nigeria [6] found that the practices of checking food labels and expiry date were significantly different between rural and urban population. More rural respondents thought that checking food labels and expiry dates did not have a significant impact on health than urban respondents. The lower awareness of food safety among rural population might contribute to their vulnerability, especially among ethnic minorities. In China, selling expired and rotten food has been reported to be prevalent in rural villages [7,8,9]. In the Food Safety Situation Investigation Report of China 2008 [10], expired food, no food labelling and hygiene of packaged food were the top food safety concerns listed by the rural villagers.

In terms of unhealthy food consumption, high salt intake has been recognized as a critical problem due to its association with hypertension [3]. Hypertension is a major public health problem and the leading disease among populations aged 40 or above in China [11,12,13]. According to the China Statistical Yearbooks Database data [14], the 2013 prevalence of hypertension in China’s rural regions was 12.31%, which was about three-fold greater than the prevalence data previously collected in 2008 (3.85%). Furthermore, it was found that Yunnan was one of the regions with the highest prevalence of hypertension in China [15]. Hence, salt intake reduction is considered as one of the preventive measures against the increase in non-communicable diseases in China [16]. Moreover, a study [17] that investigated the salt consumption habits among 21 countries found China has the highest rate of salt consumption; 80 percent of the population in China had a mean sodium intake higher than 5 g/day (the recommended maximum salt intake for adults by the WHO [3]). In particular, hypertension prevalence in rural regions was found higher than those in urban regions [18,19]. This might be due to the suboptimal medication [20] and inadequate knowledge [21] in rural regions. Therefore, salt intake-related health issues need to be emphasized, especially health interventions to reduce salt consumption and the risk of hypertension. During disasters, a high proportion of chronic diseases such as hypertension would further increase the health vulnerability of the community [22]. Furthermore, food safety would be an important health determinant for resilience. Therefore, ensuring food safety and enhancing dietary knowledge and practices are non-negligible for health-related emergency and disaster risk management (Health-EDRM) in the household level [23].

To tackle health risk-associated food safety and high salt intake problems among China’s rural villagers, understanding villagers’ practices and promoting relevant knowledge are essential. The authors and the team have developed a 1.5-year project to: 1) understand the current practices regarding food safety and salt intake of China rural villagers, 2) to promote food safety and salt intake knowledge through delivering education interventions to China rural villagers and 3) to evaluate the effectiveness of the education interventions. The target population of this project was a subgroup of an ethnic minority group in rural Yunnan, China—the Kunge. Yunnan is an earthquake-prone province in China. Keeping reserves of packaged food is a common practice in disaster-prone communities [24,25]. This project not only aimed to enhance the food-related health status of the community, but also to increase their capacity for Health-EDRM in rural community. This case study presents the background, methods, and findings of the project.

### Project Background and Study Context: Kunge People in Xishuangbanna Dai Autonomous Prefecture in Yunnan

In 2009 the Ethnic Minority Health Project (EMHP) was established in China by the authors and the team, with a core mission to develop and evaluate effective interventions of bottom-up Health-EDRM for vulnerable populations in remote areas of the country. One of the 14 project sites, Xishuangbanna Dai Autonomous Prefecture in Yunnan, was chosen to be one of the study areas on the basis of studying the risk of disaster and health situation which affected the ethnic minority group. The Yunnan ethnic minority health project was initiated in 2014.

The Kunge people reside in Kunge Mountain, Mengyang township, Jinghong city, Xishuangbanna Dai Autonomous Prefecture in Yunnan. It is one of the Xishuangbanna’s ethnic groups, officially classified as a branch of the Bulang ethnic group by the People’s Republic of China (PRC), and accounts for no more than 2500 residents in China [26]. There are total six Kunge groups in a Mengyang township with a total population of around 1600 [27]. To the authors’ knowledge, there were only a few articles exploring the Kunge population and those all were related to the language [26,27]. Bulang people have their own dialogue and religious beliefs. Males and females made up a similar proportion of the village population. Most of the population were farmers, mainly growing rice, sweet corns, tea, and rubber. The average annual household income was approximately RMB 3000–4000 (approximately USD 1.34–1.79 per day, which is lower than the official poverty line of USD 1.90 per day, defined by the World Bank 2015 [28]). Their income mainly comes from exporting agricultural products to other areas in the region. The villagers generally had a regular pattern of life and spent most of their daytime working on farms. In the estimation from our team field visit, there were 140 households with 608 residents living in ManBangTang Village in 2016.

A total of three visits were made in 2014, 2015 and 2016, respectively. A needs assessment was performed in 2014. Although agricultural activity provides the main food source for the Kunge people, as per our interviews in the 2014 visit, all villagers reported the practice of purchasing packaged food, supplied from the local food companies and markets in Mengyang township. Besides packaged food, consumption of salt-cured food, pickled vegetables, and salted meat were also reported. During the needs assessments in ManBangTang, villagers were found to use a generous amount of salt during cooking.

Based on the findings in the literature review and needs assessment conducted in November 2014, two interventions, targeting food labelling in January 2015 and salt-intake reduction in March 2016, were conducted in the same population.

## 2. Methodology

This was an 18 months serial cross-sectional survey-based study. A total of three research visits were done throughout the project period from November 2014–March 2016.

### 2.1. Sampling and Study Subject

Representatives of village households (age 18 years or older) were invited for the needs assessment (2014) and the interventions (2015 and 2016). Only one participant was invited from each household. Due to the lack of community information such as household maps, convenience sampling was used for recruiting respondents for the needs assessment and interventions in the village. Recruitment was promoted through household visits, broadcasting promotion messages by the village head, delivering leaflets, and poster distribution. There were 140 registered households with 608 residents in the ManBangTang village during the study period. There were 52 respondents (37.41% of all households) in the needs assessment (2014); 40 (25.15% of all households) participated in the first intervention in 2015 and 45 (28.13% of all households) participated in the second intervention in 2016.

### 2.2. Evaluation Tool and Data Collection

The 2014 health needs assessment tool was a questionnaire combining eight health topics (including chronic disease management, food and cooking safety (including food labelling and salt intake), personal hygiene, water supply and quality, waste management, disaster risk reduction, first-aid training, and local climate change), sociodemographic details and environmental assessments were surveyed to collect the perception and practices of the villagers. The needs assessment were developed based on the guidance report from the WHO for building a healthy village [29].

For the food labelling (2015) and salt-intake reduction intervention (2016), pre- and post-intervention questionnaires assessing knowledge, attitude, and practices (intention to practice) (KAP) of the relevant topics and sociodemographic information were used for intervention evaluations. The needs assessment results were intended to also be used to design intervention. Thus the questionnaires also included the messages delivered in the interventions (knowledge, attitude and practice (KAP)). Interventions content were adopted from relevant sources: for the salt intake standard, it was adopted from the Chinese Nutritional Society [30]; the food label standard was referenced to the Standard for the Labeling of Prepackaged Food of the China Food and Drug Administration [31]. Table 1 shows the food-related health questions in this study.

Translators were recruited to facilitate communication with the study respondents who could not speak Mandarin (6.25% out of the attendees in the second visit). Verbal consent was obtained from all study respondents prior to administering the questionnaire. All questionnaires were administered face-to-face by field researchers and local translators.

### 2.3. Statistical Analysis

The data were double entered by trained research staff. Descriptive analysis of sociodemographic information of study subjects was generated and compared to the 2010 Yunnan population census. Chi-squared test was used to compare results from pre- and post-intervention questionnaires for the effectiveness of health interventions. The significant level of this study was set at *p* < 0.05. All statistical analyses were conducted using SPSS version 21.0 (IBM Corp., Armonk, NY, United States). Ethics approval was obtained from the Joint Chinese University of Hong Kong- New Territories East Cluster Clinical Research Ethics Committee.

## 3. Results

Results are presented in three sections. The first section describes the sociodemographic characteristics of the respondents. The second part presents the results of 2014 needs assessment which included perception and practice of food labelling, general salt intake, and other food safety issues were described. The third section examines the effectiveness of the education intervention in 2015 and 2016 which targeted food labelling and salt reduction respectively.

### 3.1. Sociodemographic Characteristics of the Sample among the Three Visits

A total of 52 (37.41%), 26 (18.57%) and 34 (24.29%) households were recruited in the 2014 needs assessment, 2015 food labelling intervention and the 2016 salt-intake reduction intervention, respectively (Table 2). The sample in 2015 was younger than the population recruited in 2014 and 2016 (*p* < 0.05), while the proportion of respondents with lower education level was higher in the 2014 sample. The ethnic minority samples were not comparable to the general Yunnan population (*p* < 0.05). The study sample had a high proportion of female and people with lower education level.

### 3.2. General Food-Health-Related Practices and Experiences

#### 3.2.1. Food Labelling

Among the 52 respondents, 36 of them (69.23%) reported the practice of buying packaged food. Snacks (52.8%, 19/36) and meat (33.3%, 12/36) were the most commonly brought packaged foods. As for the practice of reading the food labels before consuming packaged food, 60.0% (21/35) reported that they would read the food label. Among this group, 37.1% (13/21) claimed they always read the labels. The expiry date was the type of information on food labels that most respondents paid attention to; 95.2% among those that would read food labels would pay attention to expiration dates. Among those who did not practice reading food labels (14/35), about half of them (6/14) stated that they did not understand the label. Among respondents who practiced reading food labels on packaged food (*n* = 21), half of them (11/21) reported seeing confusing food labels before. About half (10/21) would ignore the label if they could not understand it.

#### 3.2.2. Salt Consumption

Out of 52 respondents, 39 had reported their daily salt consumption. Among these, 56.8% (21/39) claimed they consumed one teaspoon or more of salt per day, only 27.0% (10/.39) claimed they consumed less than one teaspoon of salt per day.

#### 3.2.3. Food Safety Problems

Out of the 52 respondents, 42 answered the question “had you encountered any food safety problem before” (Table 3). Among them, 23.8% (10/42) had encountered food safety problems before. Among those had encountered food safety problems, 40% were related to expired food (4/10) while 50% were associated with food quality problems (5/10). Thirty percent (3/10) had reported feeling sick or suffering from food poisoning as a result of the food quality problem.

The most common methods used by the respondents to determine whether the food had gone bad or not were checking the appearance (42.9%, 21/44), followed by tasting (16.3%, 8/44), smelling (12.2%, 6/44), personal feeling (12.2%, 6/44) and reading the food label (6.1%, 3/44).

#### 3.2.4. Associating Factors of the Food-Health-Related Practices (Food Labelling and Salt-Intake Reduction)

The association between sociodemographic characteristics and the practice of reading food labels and salt consumption were explored using the needs assessment data (2014). The chi-squared test showed that higher education level was associated with more frequent use of reading food label (*p* = 0.046), where 80% of those with secondary education; 59.1% of those with primary education and 0.0% of those receiving no formal education would always or sometimes read the label. Age and gender were not shown to be associated with the practices of reading food labelling or salt consumption related behavior.

### 3.3. Evaluation of Health Education Intervention Effectiveness

There were 26 (81.25%) valid pre-and post-intervention questionnaires in the 2015 health intervention on food labeling topic and 40% (*N* = 18) valid pre- and post-intervention questionnaires in the 2016 health intervention on the salt-intake reduction topic. The dropout rate was due to the open-to-public format of the interventions. Some respondents left before completing the post-questionnaire or arrived after the intervention had started. Only those who had completed a pre- and a post-questionnaire were considered attending the whole intervention. This group of respondents could reflect the interventions’ effect better and therefore were considered in the evaluations. The pre- and post-questionnaires were compared using pairwise McNemar’s test to evaluate the changes in knowledge, awareness, and behaviors on both topics after the intervention. Table 4 shows the evaluation results of both interventions.

#### 3.3.1. 2015 Health Intervention on Food Labelling

In the 2015 health intervention, 26 valid sets of pre- and post- questionnaires were analyzed. Ten questions, including the food labelling awareness, knowledge, and behaviors, were asked (Table 4). Significant improvements in knowledge and awareness towards food labelling were observed after the intervention (four out of five questions). After the intervention, more respondents understood expired food could affect health (from 73.1 % to 100%, *p* < 0.001, McNemar’s test). Three food label samples were presented to test if the respondents were able to tell whether the food was expired based on the information on the food label. Significant improvements were found in two out of the three questions after the interventions (Table 4). Changes in attitude towards reading food labelling were also observed after health intervention (two out of four questions). The number of respondents who indicated that they would check the expiry date before buying and consuming packaged food increased from 34.6% to 80.8% (*p* = 0.002, McNemar’s test) and 30.8% to 76.9% (*p* = 0.002, McNemar’s test), respectively.

#### 3.3.2. 2016 Health Intervention on Salt-Intake Reduction

In the 2016 visit, 18 sets of valid pre- and post-questionnaires were collected (Table 5). Significant improvement was found in three out of six awareness and knowledge questions after the intervention. The proportion of respondents who knew high salt intake would cause hypertension increased from 50% to 88.9% (*p* = 0.039, Chi-square test). More respondents (from 44.4% to 88.9%) were aware that hypertension might cause heart disease (*p* = 0.012, Chi-square test). Although only half of the respondents could tell the correct suggested amount of daily salt consumption after the intervention, the improvement was statistically significant (from 11.1% to 50%, *p* = 0.039, Chi-square test).

After the intervention, the proportion of respondents who understand the function of the salt-reduction spoons and bottles increased from 11.1 % to 83.3% (*p* < 0.001, Chi-square test) and 11.1% to 72.2% (*p* = 0.001, Chi-square test), respectively. No significant changes were found for the attitude towards cutting down the daily salt-intake to the suggested standard or decreasing cured food consumption were observed.

## 4. Discussion

This study examined the behavioral patterns in the 2014 needs assessment (including reading food labels and salt consumption), the effectiveness of health education interventions on food labeling (in 2015) and salt-intake reduction (in 2016) among an ethnic minority-based community (the Kunge population) in rural China. Results indicated about 60% of respondents who consumed packaged food would read the food label and most of them read the food label for expiry date information. Those who had a higher education level were more likely to read the food label. However, about half of them had found food labels confusing to comprehend. About half of the respondents also reported a daily salt intake level higher than the WHO’s recommended amount [3] and about 24% of respondents reported experiencing some form of food safety problems. For both food labelling and salt-intake interventions, significant improvements in knowledge, including the health effects of consuming expired food, how to read the expiry date in a food label, the suggested amount of daily salt intake for adults, the association among high salt intake, hypertension and heart diseases, and the function of the salt-restriction-spoon and bottle, were observed after the interventions. In terms of attitude towards behaviors, more respondents indicated that they would check the expiry date before buying and eating packaged food and would control their salt consumption after the interventions, though the changes in attitude for salt consumption control was not statistically significant.

Only 60% of the respondents who had bought packaged food would pay attention to the food label. This figure was lower compared to another similar research from India. The study from Puducherry in India [33] reported that 90% of the respondents would pay attention to the food labels on packaged food. The relatively lower rate found in the Kunge respondents might be associated with the lack of relevant health education and education level in the community. As mentioned in the introduction, there are many incidents of problematic food products in rural areas [7,8,9]. This suggested that the awareness of food safety was insufficient in these areas, which increases the vulnerability in this population. Our needs assessment’s result found that respondents with a higher education level had greater awareness of reading food labels. This supports the need, and potential value, to raise the food-health-related awareness in the community. The evaluation results showed that education interventions were effective in increasing awareness as well as their attitude towards the corresponding behaviors. A larger scale of education programmes and evaluation of the long-term impact of such programmes should be considered for the community.

High salt intake appeared to be common in the Kunge population in Yunnan. Nearly 60% of the respondents consumed one teaspoon or more per day, which exceeded the suggested standard for adults by the WHO (under one teaspoon) [3]. The result was consistent with those found in studies of other minority groups from rural China communities [16,17]. High salt intake was also found in another ethnic minority, the Yi, in Yunnan [34]. About 80% of them preferred preserved food which was suggested to be one of the underlying causes of a high prevalence of hypertension (37%) among the Yi population. Hence, salt-intake reduction should be promoted in rural community in Yunnan, in particular for ethnic minority communities, to reduce the risk of relevant health problems.

Among those who have encountered food safety problems (23.8%), food spoilage (50%) and expired food (40%) were the top two food safety problems reported. This finding is different from that of a study conducted in urbanized regions, Beijing and Nanjing, in China [35]. The greatest concerns found in the urban study were about food hygiene and food poisoning while expiry date only ranked the 4th among the food safety issues. The differences in food safety concerns between rural and urban regions may be related to the food surveillance systems as spoilage food was more commonly found in the food markets in rural areas due to the less efficient surveillance of food quality [7].

Significant improvement in knowledge and intention to practice the behavior were identified in both the salt reduction and food labelling interventions. Despite the improvements in the understanding of the effects of consuming expired food and the ability and willingness to read the food label, the proportion of respondents that claimed they would not purchase or consume expired food (76.9%) was still lower than that reported in another Chinese study conducted in 31 provinces (89.4%) [36]. This may be related to the economic status among the rural village. Our needs assessment in 2014 found that the study community was under the poverty line. For financial reasons, respondents might still consume expired food even if they understood the potential adverse health effects. Improving the quality of local food supply and encouraging the community to purchase packaged food when needed (to avoid overstocking) might help to reduce the chances for the community to consume expired food. More studies are warranted for drafting effective policies to tackle the problem. Similarly, improvement in salt intake-related knowledge did not imply a change of behavior in salt-intake and consumption of cured food. More research will be needed to understand the economic, cultural, and behavioral challenges.

There were a few limitations in this study. First, only immediate effects of the interventions could be measured due to the project nature. Evaluation of long-term effects should be considered whenever possible to assess and achieve sustainability of the knowledge transferred. Second, this study did not cover the entire food-related behavior topic. Among food safety and consumption pattern, only salt consumption and food labelling of expiration date were investigated. There are other important dietary behaviors such as sugar and oil consumption and food-safety related behavior like storage or information seeking [5] to be explored in future studies. Third, the sample size for evaluating the effectiveness of the intervention was small which may not represent the whole community, especially for the salt-intake reduction. This limited the power of analysis and did not allow subgroup analysis and special consideration should be paid when interpreting the results for scaling up of related programs. Furthermore, there is also a lack of published or official documentation about this minority group for data triangulation. Lastly, language barrier was another limitation. For that, translators were invited to assist during data collection to minimize the barrier. Yet, despite of these limitations, this article will provide information about food and diet, two important aspects of H-EDRM for this special minority group in China.

## 5. Conclusions

This study identified the food-related Health-EDRM issues in a rural ethnic minority (Kunge) village in Yunnan, China. Not checking expiry dates of packaged food and a high salt diet were the most common problems in the Kunge community. Education interventions were found effective in improving food-related health knowledge despite the small sample sizes. These included the adverse health effects of consuming expired food, how to read the food label for expiry date information, the association among high salt intake, hypertension and heart health, the suggested maximum amount of salt intake for adults, and the function of salt-restriction-spoon and bottle. These interventions were also shown to be effective in changing attitudes toward some behaviors, such as willingness to read the food labels before buying and consuming packaged food. However, no significant improvements were found for the attitudes toward not consuming expired food, control salt-intake and decreased consumption of cured food. This suggests that although health education was effective in raising awareness and promoting health knowledge in a rural ethnic minority community, other elements, including economic status, culture and local food policy, should be considered when promoting changes of health behavior [37]. Special attention should be paid when interpreting the results for the representativeness of the small sample size.

## Figures and Tables

**Table 1:** Questions used in assessing food labelling and salt consumption awareness, attitude and behaviour in the visits (2014, 2015 & 2016).

2014 Visit: Needs Assessment	2015 Visit: Food Labelling	2016 Visit: Salt Consumption
Food labelling related information	Pre- & post- intervention question	Pre- & post- intervention question
Have you ever brought package food before? (exclude rice, oil, and salt) (Answer: Yes/ No)	Do you agree: “I understand that consuming expired food will affect my health.”? (Answer: Yes/ No)	Do you think you consume enough salt per day? (Answers: Not enough, enough, more than enough)
What kinds of packaged food you usually buy? (Answers: multiple choice with various food type)	Do you agree “I can distinguish whether the food is expired or not.”? (Answer: Yes/ No)	Do you think high salt consumption will cause hypertension? (Answer: Yes/ No)
How often do you read the food label on packaged food before consuming it? (Answer: Always, sometimes and never)	Is the following packaged food expired? (3 Yes/No questions with their local food package images)	Do you think cure food is high in salt? (Answer: Yes/ No)
Why don’t you check food labels on packaged foods? (Answer: Multiple choices)	Do you agree “I will check the food label before purchasing the packaged food.”? (Answer: Yes/ No)	Do you think hypertension causes cardiovascular disease? (Answer: Yes/ No)
What information on the food label will you usually pay attention to? (Answer: Multiple choices)	Will you check the food label before consuming the packaged food? (Answer: Yes/ No)	Do you think hypertension causes stroke? (Answer: Yes/ No)
Have you ever encountered any food label that you didn’t understand? (Answer: Yes/ No)	Do you agree “I will not purchase and consume any expired packaged food.”? (Answer: Yes/ No)	Do you know the recommended daily salt consumption level for adults? (Answer: Yes/ No with an open answer on the salt amount)
If you encounter a food label that you don’t understand, what will you do? (Answer: Multiple choices)	I will remind my family and friends to check the food labels and pay attention to expired food. (Answer: Yes/ No)	Do you know the function of a salt restriction spoon? (Answer: Yes/ No)
Salt-consumption related question	Post-intervention only question	Do you know the function of a salt restriction bottle? (Answer: Yes/ No)
Please estimate your daily salt consumption. (3 answers: less than, approximately and more than 1 teaspoon)	Do you agree “I am confident to build a habit of checking the food labels on packaged food.”? (Answer: Yes/ No)	Can you control your daily salt consumption at less than 6 g in the next month? (Answer: Yes/ No)
Food safety related questions		
Have you experienced any food safety problem before? (Answer: Yes/ No)		Can you consume less cure food in the next month? (Answer: Yes/ No)
Have you got sick or suffered from food poisoning after experiencing the above food safety problem? (Answer: Checklist)		
Which factor will you consider to determine whether the food has gone bad or not? (Answer: Multiple choices)		

**Table 2:** Sociodemographic characteristics of the households participating in the 2014 to 2016 field visits in ManBangTang Village, Yunnan, China.

	ManBangTang Village			Yunnan ^a^
	2014	2015	2016	2010
*N*	52	26	34	NA
Age *	30.0 (24.25–40.0)	20.0 (18.0–37.25)	35.0 (24.0–48.0)	NA
Female	20 (38.5%)	13 (43.3%)	11 (34.4%)	48.11%
Male-to-female ratio	1:0.63	1:0.76	1:0.52	1:0.9273
Education level *				
Illiterate/Non-formal Education	7 (13.7%)	3 (11.5%)	9 (26.5%)	14.97%
Primary school	30 (58.8%)	6 (23.1%)	10 (29.4%)	43.39%
Junior secondary	13 (25.5%)	17 (57.7%)	14 (41.2%)	27.48%
Senior secondary	1 (2.0%)	2 (7.7%)	1 (2.9%)	8.38%
Tertiary	0 (0.0%)	0 (0.0%)	0 (0.0%)	5.78%

^a^ Sources: the Sixth National Population Census report in 2010 [32]; * *p* < 0.05.

**Table 3:** Needs assessment results related to food labelling, salt consumption, and food safety problems.

Questions (*N*)	Items/Choices	*N* (%)
Food Labelling ^a^		
Type of food packages they bought (*n* = 36)		
	Fish	2 (5.6%)
	Meat	12 (33.3%)
	Fruit	6 (16.7%)
	Cereal	5 (13.9%)
	Milk/Product	3 (8.3%)
	Egg	2 (5.6%)
	Snacks	19 (52.8%)
	Others	4 (11.1%)
Check the food label before consuming the packaged food (*n* = 35)		
	Always	13 (37.14%)
	Sometimes	8 (22.86%)
	Never	14 (40 %)
Reasons for not checking the food labels on the packaged food (*n* = 14) ^b^		
	Do not know there is a food label on the packaged food	1 (7.1%)
	Do not understand	6 (42.9%)
	Never thought about this before	2 (14.3%)
	Don’t know	1 (7.1%)
	Rejected Answer	4 (28.6%)
Types of food labels subject pay attention to (*n* = 21)		
	Nutrition **	4 (19.0%)
	Expiration date	20 (95.2%)
	Way of Storage	2 (9.5%)
	Manufacture location	3 (14.3%)
Had you met the confusing food labels (*n* = 21)		
	Always	4 (19.0%)
	Sometimes	7 (33.3%)
	Never	5 (23.8%)
	Don’t Know	3 (14.3%)
	Refuse to answer	2 (9.5%)
What will you do if you meet any un-understandable food label (*n* = 21)		
	Ignorance	10 (47.6%)
	Ask others for information	2 (9.52%)
	Not to purchase/eat	6 (28.57%)
	Don’t know	2 (9.52%)
	Refuse to answer	1 (4.76%)
Salt consumption		
Daily Salt intake(*n* = 39)		
	1 teaspoon or above	21 (53.85%)
	Less than 1 teaspoon	10 (25.64%)
	Cannot estimate the salt intake	8 (20.51%)
Food safety related problem		
Encountered any Food safety problem (*n* = 52)		
	Yes, usually	2 (4.76%)
	Yes, but not often	8 (19.05%)
	Never	32 (76.19%)
Encountered what kinds of food safety problem (*n* = 10) ^c^		
	Food expired	4 (40%)
	Counterfeit food	1 (10%)
	Food spoilage	5 (50%)
	Usage of illegal additive	1 (10%)
	Would you get any food poisoning due to food safety problem	3 (30.0%)
Factors to define whether the food is gone bad or not (*n* = 44)		
	Taste	8 (16.3%)
	Smell	6 (12.2%)
	Appearance	21 (42.9%)
	Feeling	6 (12.2%)
	Date/Food Label	3 (6.1%)

** One respondent specifically mentioned protein and fat; ^a^ Respondents were also asked if they had bought Cereal and Beans, but none replied they had; ^b^ No respondent replied “No label on the packaged food” and “not important” as the reason for not checking the food label; ^c^ No respondents report they had encountered “wrong/misleading instruction” as a food safety problem.

**Table 4:** Key findings on the effectiveness of pre-and post-intervention in the 2015 Food Labelling.

2015 Health Intervention on Food Labelling (*N* = 26)			
	Pre- (%)	Post- (%)	*p* value
Food Labelling awareness and knowledge			
Understand that eating expired package food will affect health	73.1%	100.0%	0.004 *
Able to distinguish if the food is expired	46.2%	88.5%	<0.001 *
Question sample 1 (Able to distinguish if the food is expired)	36.0%	72.0%	0.012 *
Question sample 2 (Able to distinguish if the food is expired)	44.0 %	48.0%	0.780
Question sample 3 (Able to distinguish if the food is expired)	24.0%	54.0%	0.023 *
Food Labelling behavior			
Will not eat or buy any expired food	69.1%	76.9%	0.532
Check the expiry date every time before buying	34.6%	80.8%	0.001*
Check the expiry date every time before consuming	30.8%	76.9%	0.001 *
Remind my family and friends keep checking the expiry date	69.2%	92.35	<0.035 *
Confident enough to cultivate the habits of reading food labels	-	100%	-

* *p* < 0.05.

**Table 5:** Key findings on the effectiveness of pre-and post-intervention in 2016 Salt Reduction.

2016 Health Intervention on Salt Reduction (*N* = 18)			
	Pre- (%)	Post- (%)	*p* value
Salt reduction awareness and knowledge			
Consume enough salt on a daily basis	61.1%	55.5%	1.000
High salt diet cause hypertension	50%	88.9%	0.039 *
Cured food contains high salt amount	61.1%	72.2%	0.375
Chronic hypertension cause heart disease	44.4%	88.9%	0.012 *
Chronic hypertension cause stroke	38.9%	72.2%	0.065
Understand the suggested amount of daily salt consumption	11.1%	50%	0.039 *
Salt reduction behavior			
Understand the function of salt-restriction spoon	11.1%	83.3%	<0.001 *
Understand the function of salt-restriction bottle	11.1%	72.2%	0.001 *
Control the consumption of salt below 6g daily in future month	66.7%	94.4%	0.063
Control the consumption of cured food in future month	72.2%	94.4%	0.125

* *p* < 0.05.
